# International ring trial to validate a new method for testing the antimicrobial efficacy of domestic laundry products

**DOI:** 10.1371/journal.pone.0269556

**Published:** 2022-06-03

**Authors:** Toni Monleón-Getino, Michele Cavalleri

**Affiliations:** 1 Department of Genetics, Microbiology and Statistics, Section of Statistics, University of Barcelona, Barcelona, Spain; 2 GRBIO, Research Group in Biostatistics and Bioinformatics, Barcelona, Spain; 3 BIOST^3^, Research Group in Clinical Statistics, Bioinformatics and Computational Biodiversity, Barcelona Spain; 4 European Committee for Standardisation, CEN/TC 216/WG 3 Food Hygiene and Domestic and Institutional Use, Brussels, Belgium; 5 Eurofins Biolab SRL, Vimodrone (Milano), Italy; University of Porto, PORTUGAL

## Abstract

Due to greater environmental awareness, domestic laundry habits are changing, and antimicrobial control by chemical methods has become an essential factor to compensate for the use of lower temperatures during washing machine cycles. Disinfectants added to laundry detergents are a preventive strategy to reduce the transmission of bacteria, fungi, and viruses in the home, correct aesthetic damage (e.g., spotting, discolouration, and staining), and control the microbial contamination that leads to malodour. In Europe, disinfectants are regulated by the EU Biocidal Products Regulation (No. 528/2012), which stipulates that antimicrobial efficacy must be evaluated according to standardized methods. Current European standards for laundry sanitization only apply to clinical settings (EN 16616: 2015) and are restricted to the main wash cycle. Therefore, there is a gap in the EU standards regarding the testing of product efficacy in household laundering. With the aim of addressing this gap, an international ring trial was organized to evaluate the robustness of a new method (prEN 17658) designed to test the efficacy of antimicrobial laundry products in a domestic setting. The seven participating laboratories were equipped with 5 different laboratory-scale devices to simulate the washing process, and they evaluated 7 microbial parameters for 2 experimental conditions and 3 levels of active substance. The analysis of data according to ISO 5725–2 and ISO 13528 demonstrated that the method was robust. All reproducibility standard deviation values were between 0.00 and 1.40 and the relative standard deviation indicates satisfactory reproducibility. Values of logarithmic reduction ranged from less than 2 log_10_ for tests with water to more than 5 log_10_ when disinfectants were added. The evidence generated by the ring trial was presented in a proposal for a standardized method under CEN/TC 216, in which the SOP used in the ring trial is referred to as the prEN 17658 phase 2 step 2 test method covering chemothermal textile disinfection in domestic settings.

## Introduction

In the last decades, laundering processes have been redesigned to be more energy efficient, achieving equivalent levels of soiling and stain removal at lower washing temperatures. However, this may imply a reduced control of microorganisms [1], with a subsequent impact on health, as contaminated laundry has been identified as a significant risk factor in the transmission of certain pathogens [1]. Extending the duration of the laundry process can only partially compensate for the effect of lower temperatures, and is not always feasible in domestic settings [2]. Another issue for consumers is malodour control, as it has been demonstrated that if bacteria are not adequately removed or killed, they will remain on fabrics and cause bad odours or damage by metabolizing sweat compounds ([1–5]). One option to reduce bacterial levels during the laundry process, especially at low temperature (<40°C) and short cycles (<60 min), is the addition of disinfectants. Biocidal products must be registered prior to commercialization and therefore standardized test methodologies simulating their use are required. Although in Europe there is an existing standard method (EN 16616:2105) [6] to evaluate the antimicrobial efficacy of detergents in clinical settings, there are no standards for the domestic area. In the USA, the standards ASTM 2274 [7] and ASTM 2406 [8] are specific for the domestic environment, whereas EN16616 applies only to areas and situations where disinfection is medically indicated; moreover, it does not include a test setup for the rinse cycle.

To address this gap, Schages et al. [9] published an experimental setup simulating a complete laundry process compatible with a household setting. Their method uses a laboratory-scale washing device with a profile similar to a typical domestic washing machine, eliminating uncontrollable factors such as water volume and mechanical action, so the results are more comparable. Not only does the method allow products to be tested throughout the laundering process (including the rinse cycle) but can also systematically assess the impact of modifying a range of parameters (e.g., active ingredients or different types of textiles). However, it might be necessary to separate the typical program phases in terms of the application of parameters and passing criteria.

Textile disinfection falls within the scope of the EU Biocidal Products Regulation (No. 528/2012). This legal framework for products with a biocidal effect includes multiple methodologies to build evidence for product efficacy. Standard protocols are preferred for such processes, but they cannot measure all the efficacies attributed to a product, and therefore a new method may need to be built from scratch. The conditions in which an antimicrobial product is effective strongly influences the way it will be applied and its estimated impact in terms of environmental and human risk. For effective hygienic control of domestic laundry processes, standardized methods are required to analyse the efficacy of detergents, additives, and rinse aids in combination with washing machine effects. The experimental setup of Schages et al [9] was able to demonstrate the impact of different parameters and is well-balanced in terms of human and other resources, such as time, equipment and material. Therefore, a CEN/TC216 task group studied the suitability of this experimental setup for application as an EN test protocol.

In this process, a group of experts validated the performance of the experimental setup according to CEN/TC technical requirements. A ring trial involving seven laboratories equipped with different types of laboratory-scale devices was carried out, evaluating repeatability (“Precision in measurements under conditions that include the same measurement procedure, same operators, same measuring system, same operating conditions and same location, and replicate measurements on the same or similar objects over a short period of time.” [10]), and reproducibility (“Precision in measurements under conditions that may involve different locations, operators, measuring systems, and replicate measurements on the same or similar objects.” [10]); thanks to that have challenged the SOP constituting the draft that will be sent for CEN enquiry [11] The aim of the study was to validate the robustness of the new method for the evaluation of antimicrobial efficacy of domestic laundry products.

## Materials and methods

### Participating laboratories

Seven laboratories participated in the study. A public open call for interested laboratories was launched in September 2018 through associations and the CEN/TC network. The only requirements for participation were a commitment to the timeline of the exercise and access to a suitable laboratory-scale device for the test.

### Ring trial scheme

The ring trial was structured in 4 steps: 1) training of participants and material distribution; 2) intercalibration exercise; 3) testing of rinse cycle products in simulated conditions; and 4) testing of main wash cycle products. Participating laboratories were trained in a workshop in Rhein-Waal University (September 2019), where the complete method was demonstrated, and method instructions were given. The coordinating laboratory prepared all the documentation and materials required for the exercise: standardised spread sheets for collecting raw data, and reference materials including textiles, reference strains and interfering substances from the same batches. Each step had a clear objective and was carried out within a defined time frame. At each step, raw data including deviations and comments were collected and compiled, and statistical analysis was performed. Throughout the ring trial, an exhaustive follow-up was carried out with periodic meetings, and technical consulting and support were provided for all partners.

Step 1 was carried out in Sept. 2019, step 2 between Sep.-Nov. 2019, step 3 between Dec. 2019- Jan. 2020 and step 4 between Feb.-Mar. 2020.

### Reference microorganisms

EN standard reference strains were used (*Pseudomonas aeruginosa* NCTC 13359/ ATCC 15442, *Escherichia coli* NCTC 10418/ ATCC 10536, *Staphylococcus aureus* NCTC 10788/ ATCC 6538, *Enterococcus hirae* NCTC 13383/ ATCC 10541 and *Candida albicans* NCPF 3179/ ATCC 10231). All seed cultures were purchased from the British Collection of Type Cultures for bacteria (NCTC) and fungi (NCPF).

### Reference material

Regarding the products tested, the most representative laundry biocidal active ingredients on the market were chosen for both the main wash and the rinse cycle, but they were not tested with commercialized products to avoid conflicts of interest. Likewise, products used in the main wash and the rinse cycle were calculated to the volume of one canister. For the main wash, IEC Non-Phosphate Detergent (A) (IEC-A) and detergent plus disinfectant [IEC-A, sodium percarbonate, tetraacetylethylenediamine (TAED)] were tested. In accordance with EN 60456 [12], when using a 500 ml volume, the amount of IEC-A* when testing only detergent was 3.3 g (test B), and 2.5 g IEC-A*+ 0.6 g sodium percarbonate + 0.1 g tetraacetylethylenediamine (TAED) when testing detergent with a disinfectant (test C). For the rinse cycle, dodecyl dimethylammonium chloride (DDAC) was tested at two doses: 0.04% (non-efficacious) (test E) and 0.4% (efficacious) (test F). Water controls containing 0% of product were included in the exercise for both the main wash and rinse cycle (tests C and F, respectively), investigating only the influence of the mechanical process on the microbial reduction. The concentrations of the products used were chosen to observe the transition from non-efficacy to efficacy against reference bacteria and yeast.

A textile of polyester/cotton (65% polyester/ 35% cotton,170 g/m2, ibid.) wfk 20 A (W 20 A, wfk Testgewebe, Brüggen, Germany) was used as the ballast load to simulate domestic conditions. 1 cm x 1 cm cotton fabric pieces (wfk 10 A, 100% cotton, wfk Testgewebe GmbH, Brüggen, Germany) were used as carriers. Both textiles, ballast fabrics and carriers, were desized by boiling for 30 min with distilled water twice, followed by a sterilization cycle in the autoclave and drying. In addition to the ballast textiles, when the main wash products were tested, SBL2004 (SBL2004, wfk Testgewebe, Brüggen, Germany) was added to provide a source of organic soil ballast, which is relevant for domestic applications.

### Laundering process

The tested durations were 30 min for the main wash (representing the lower limit of main wash durations in domestic washing machines) and 10 min for the rinse cycle (representing the shortest rinse cycle in domestic washing machines). In the case of the rinse cycle, the investigated temperature was 20°C, which is within the standard conditions for testing at room temperature. Regarding the main wash, 30°C was chosen as a representative temperature. In recent years, there has been a continuing trend towards lower temperatures in household laundering (≤40°C) [2]. The IFH (International scientific forum on home hygiene) recognises the need for laundering at a low temperature (30°C) to save energy [3]. Previous results indicate a reduction in effectiveness from 40°C to 30°C, but little or no further reduction from 30°C to 15°C [3]. The tests were carried out using hard water (375 mg/l CaCO3) prepared according to EN 1276 [13]. Each sample was tested under each condition in three independent runs.

## Method

### Principles of the method

**Fig 1:**
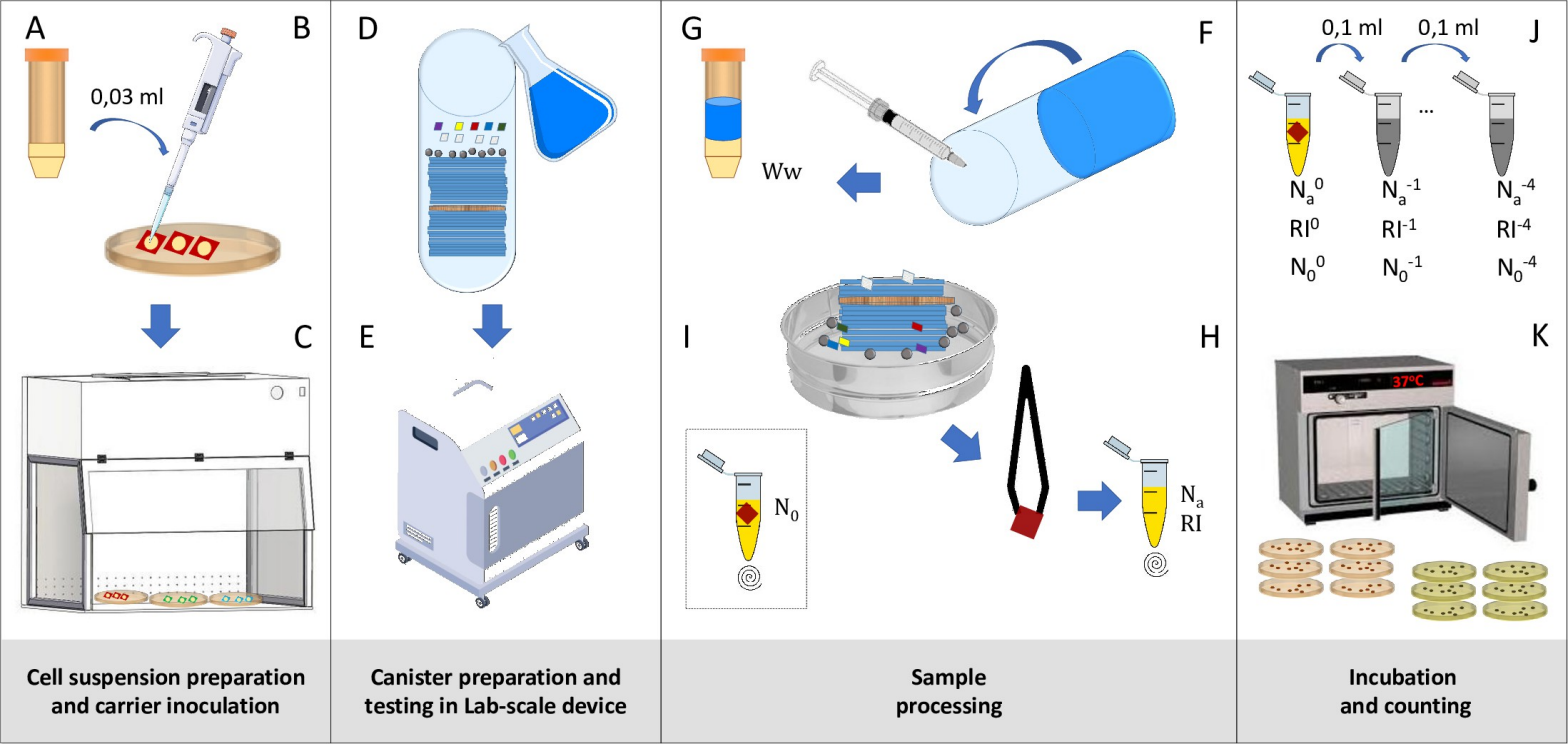
Method workflow. **A**: Cell suspension preparation for each strain tested. **B**: Carrier inoculation. **C**: Carrier drying process in the flow cabinet. **D**: Canister preparation. **E**: Testing time at the corresponding temperature in the lab-scale tumbling device. **F**: Wash water recovering and neutralizing. **G**: Content addressing. **H**: *N*_a_/*R*I carrier recovery and mixing in neutralizer. **I**: *N*_0_ mixing in neutralizer. **J**: 10-fold dilution preparation. **K**: Plate incubation and counting.

The basic prinsciples of the method have been evaluated in a previous study at Rhein-Waal University, as mentioned [9]. Briefly, the test is performed in closed 1 l canisters made of stainless steel. The canisters are then subjected to a set temperature and mechanical action in a laboratory tumbling device, as established in textile processing laboratories, e.g., RotaWash (M228C, SDL Atlas, Rock Hill, SC, USA). Typically, 8–12 canisters can be processed in parallel under the same parameters. The load in each canister consists of 100 g of ballast fabric and 500 ml of water (with or without the product in the appropriate concentration). Test microorganisms are immobilizated onto textile carriers and added to the canister. After incubation under defined parameters, the remaining active cells of the test organisms are determined compared to a water control (without product) and the logarithmic reduction factor is calculated. Based on the results of the pre-study [9], the test protocol was outlined as follows: 1) use reference organisms from EN 16616 (*Pseudomonas aeruginosa*, *Escherichia coli*, *Staphylococcus aureus*, *Enterococcus hirae* and *Candida albicans*); 2) use polyester/cotton as the ballast load; 3) establish working cultures according to EN 13697 followed by resuspension of the pellet in 0.3 g/l BSA solution (bovine serum albumin); 4) contaminate carriers on the surface; and 5) carry out water counts by direct plating. After the selected parameters were confirmed as feasible by three of the laboratories (data not shown), the final ring trial protocol was defined as described below. Fig 1 shows the workflow with the main steps of the method.

#### Preparation of the carriers

For each test strain, at least two contaminated carriers per run were needed. The working cultures were prepared according to EN 13697 [14]. Subcultures from the stock culture were performed by streaking onto plates containing tryptone soy agar (TSA) or malt extract agar (MEA) and incubated in the appropriate conditions [(37 ± 1)°C/24h or (30 ± 1)°C/48h for bacteria and yeast, respectively]. After the incubation period, a second subculture was prepared in the same conditions. Loopfuls of the second subculture were transferred to a diluent and the organism stock was adjusted to above 1.5 x10^9^ cfu/ml for *P*. *aeruginosa* and *E*. *coli*, above 1.5 x10^8^ cfu/ml for *E*. *hirae* and *S*. *aureus*, and above 1.5 x10^7^ cfu/ml for *C*. *albicans* followed by centrifugation and resuspension in 0.3g/l BSA. Subsequently, the carriers were inoculated with 30 μl of the corresponding cell suspension to ensure a recovery between 6.15 log_10_ and 8.15 log_10_ for bacteria and between 5.15 log_10_ and 7.15 log_10_ for yeast. The carriers were then placed in sterile petri dishes and dried in a biological safety cabinet, preferably for no longer than 30 min. All values of control carriers (*N*_0_) taken into account are listed in S6 Table (Annex).

#### Preparation of the testing canister

The amount of ballast load, soil ballast, and detergent was calculated to the 500 ml volume of water in one canister of the laboratory-scale washing machine. For the main wash cycle, ballast fabric (100 g/canister) was used, consisting of 96.5 g of sterile ballast fabric and 3.5 g of textile comprised of SBL2004 swatches. For the rinse cycle, only 100 g of sterile ballast fabric was used, as clean conditions were tested. Added on top of the ballast load were 8 sterile metal beads to simulate the mechanical effect of the washing machine, 5 contaminated carriers (one for each microorganism tested) and 4 sterile carriers for evaluating the cross-contamination. S1 Fig shows the canister distribution in the main wash and rinse cycles.

#### Test procedure

500 ml of water of standardized hardness containing the diluted test product was added to the canister, which was placed in the laboratory-scale tumbling device for the testing time at the corresponding temperature. After the contact time, 15 ml of wash water was recovered with a syringe and mixed with 15 ml of a suitable double concentrated neutralizer (see S5 Table). Efficacy of the neutralisers was established in accordance with EN 1276 [13]. Afterwards, 2 ml of the 1:1 mixture was plated onto 5 TSA plates and 5 MEA plates (2 ml per plate) and incubated in specific conditions (TSA at 37°C for 24h and MEA at 30°C for 48h). All carriers [5 contaminated carriers (*N*_a_) and 4 non-contaminated carriers (*R*I)] were recovered from the ballast load and each was added individually to a tube containing 1 ml of neutralizer. Each tube was mixed for 10 min at 15°C– 18°C and 1 000 r/min using a mixer to recover any remaining microorganisms. For counting, 10-fold dilutions to 10^−4^ were prepared using a diluent. Samples of 0.4 ml of each dilution were plated on the corresponding agar plates in duplicate (TSA for bacteria and MEA for *C*. *albicans*). To evaluate cross-contamination, the eluate of two non-contaminated carriers were plated in TSA, and the eluate of two non-contaminated carriers in MEA. The value of the non-treated carriers (*N*_0_) for each test series was used as a basis for calculating the reduction after exposure.

### Data collection and treatment

Each laboratory was provided with a spreadsheet to collect data. This document includes laboratory information (room temperature/ humidity, technician), product information (appearance, batch number, store conditions), experimental conditions (plating type, neutralizer used, diluent, test temperature, test contact time, incubation temperature, incubation time), and raw data of each parameter evaluated (number of cfu counted per sample of test suspensions, carrier control, carriers after laundering, cross-contamination of carriers, and wash water). Datasheets from participants were collected and processed by the coordinating laboratory for statistical analysis.

### Determination of cell counts on carriers and in the wash water

To determine the microbial load of the carriers, tests were run in triplicate. After laundering, the microbial count on the test carriers was determined in the same way. To check for cross-contamination, four sterile carriers (RI) were laundered together with the contaminated carriers and processed in the same way as *N*_0_ and *N*_a_. The numbers of cfu/cm^2^ on plates were used to calculate the microbial load in the extraction liquid:

$$N_{0}/N_{a}/RI=\frac{c}{0,4x(n_{1}+0,1\times n_{2})d}$$ (Eq 1)

*N*_0_ is the number of cells per carrier on one untreated carrier; *N*_a_, the number of cells per carrier in the test mixture at the end of the contact time; *R*I, the number of survivors in the four non-contaminated carriers; *c*, the sum of VC-counts taken into account (number of cfu counted per 0.4 ml of sample); n_1_, the number of VC-counts taken into account in the lower dilution; n_2_, the number of VC-counts taken into account in the higher dilution; *d*, the dilution factor used; and 0.4 is the volume sample in ml per plate.

Furthermore, wash water (*W*W) was investigated to determine the transfer of microbial cells to the water:

$$ww=\frac{c}{n}$$ (Eq 2)

*W*W is the number of survivors in the wash water; c, the sum (five plates) of the VC-counts taken into account; and *n*, the number of VC-counts taken into account in the direct dilution.

Plates with fewer than 14 cfu or more than 330 cfu were not considered. If the count after laundering in the laboratory-scale washing machine was lower than 14 cfu/plate in the direct dilution, *N*_a_ was set to 14 cfu/plate. When the VC-values were equal to zero or below 14, the final *N*_a_ value was recorded as the detection limit value: 1.54 log_10_ (*N*_a_, *R*I) and 1.15 log_10_ (*W*W). In these cases, logarithmic reduction (LR) values were calculated by extracting the value of the detection limit from *N*_0_.

### Calculation of reduction factors

In this method, the LR is considered as a combination of inactivation and detachment of microbial cells. Following EN standards, the LR (Eq 3) is the difference in the common logarithm of the microbial count per mL of the initial load in the carriers before (*N*_0_) and after laundering (*N*_a_).


$$LR=N_{0}‐N_{a}$$ (Eq 3)

### Statistical analysis and methods

Compiled data sheets were sent to Monleón-Getino for the statistical analysis and to check the repeatability and reproducibility of the method according to ISO 5725–2 [15] and ISO 13528 [16]. ISO 5725 refers to the accuracy (trueness and precision) of measurement methods and results. The second section (5725–2) refers to a basic method for the determination of repeatability and reproducibility of a standard measurement method. ISO 13528 refers to the statistical methods used in proficiency testing by interlaboratory comparison and provides detailed descriptions of statistical methods for proficiency testing providers to use to design proficiency testing schemes and to analyse the data obtained from those schemes.

In order to compute repeatability and reproducibility in the current interlaboratory study, a mixed linear model [fixed and random factors [17]] was used according to Flores et al. (2018) [18]. This model can be described in the following way:

$$Y_{ij}=\mu +B_{i}+\varepsilon_{ij}$$ (Eq 4)

where *Y*_*ij*_ is the response variable (quantitative variable of interest in the experiment obtained by laboratories for different parameters, e.g., LR-PA, *W*W-TSA), μ is the mean value of the response variable, *B*_*i*_ is a random variable with normal distribution [*B*_*i*_~*N*(0, *σ*_*B*_)] that represents the variation between laboratories, and *ε*_*ij*_ is a random variable with normal distribution [*ε*_*ij*_~*N*(0, *σ*_*r*_)] that represents the repeatability; it is assumed that *ε*_*ij*_ and *B*_*i*_ are independent. Thus, in the normal model it is observed that

$$Y_{ij}∼N(\mu ,\sigma_{R})$$ (Eq 5)

where *Y*_*ij*_ is the response variable, μ is the mean value of the response variable and *σ*_*R*_ is the total process variability computed from $\sigma_{R}^{2}=\sigma_{B}^{2}+\sigma_{r}^{2}$, where $\sigma_{R}^{2}$ is the total variability (reproducibility variance), $\sigma_{B}^{2}$ is the between-laboratory variance, and $\sigma_{r}^{2}$ is the repeatability variance (residual variance) [18].

From a statistical point of view, model (Eq 3) can be tested statistically using an analysis of variance (ANOVA) method if $H_{o}:\;\sigma_{B}^{2}=0$; in the case *p*−*value*<*α* we can conclude that $\sigma_{B}^{2}>0$ and consequently $\sigma_{R}^{2}=0+\sigma_{r}^{2}$.

In the current study, simplified notation will be used for the different estimates of the variabilities calculated using model (Eq 3), as follows: $SR=\sqrt{{\hat{\sigma }}_{r}^{2}+{\hat{\sigma }}_{B}^{2}},\;Sr={\hat{\sigma }}_{r}$ and $S_{R}/S_{r}={\hat{\sigma }}_{B}/{\hat{\sigma }}_{r}$

Relative standard deviation (RSD) was also calculated. RSD is defined as the deviation measurement indicating the different numbers in a particular data set scattered around the mean. This formula shows the spread of data in percentage and is computed:

$$RSD=100*\frac{SR}{Mean}$$ (Eq 6)

The ratio between repeatability and reproducibility standard deviation (S_R_/S_r_) is defined as the ratio between repeatability and reproducibility standard deviations and evaluates if there are minor or systematic differences between the laboratories [19].

The R package [20] was used to carry out all the numerical calculations in the present investigation. To obtain the decomposition of the variability according to the indicated model (Eq 3), the aov and lmer functions of the basic and lme4 libraries [21] were used. aov and lmer functions fit an ANOVA model using linear models for each factor involved in the experimental design, but aov uses ordinary least squares (OLS) estimation [22], and lmer uses restricted maximum likelihood REML (a version of the maximum likelihood (ML) method) [23].

Other statistical procedures were used in the current study: the Cochran test [24,25] to detect abnormal variability and Grubbs test [26] to detect outliers in the observations for each variable. Englemann.Hecker.Plot.Algorithm function of the R library Diagnobatch [27] was also used.

## Results and discussion

Using an ANOVA model (see Eq 4), the standard deviation associated with repeatability, reproducibility, and between-laboratory differences was analysed. The statistical results for the main wash and rinse cycles are summarized in Tables 1 and 2, respectively. All product concentrations were tested on separate days in triplicate. Lab 6 could not perform the rinse cycle tests. Therefore, the total samples analysed were N = 63 for the main wash and N = 54 for the rinse cycle.

**Table 1:** Precision statistics for testing per prEN 17658 in the main wash conditions.

	VARIABLE	Mean (CI95%)	S_R_	S_r_	RSD %	S_R_/S_r_
TEST A	LR PA	2.37[2.026, 2.714]	0.77	0.58	32.07%	S_R_<2·S_r_
	LR EC	1.79[1.534, 2.048]	0.59	0.31	31.28%	S_R_<2·S_r_
	LR SA	1.31[1.097, 1.527]	0.48	0.36	35.88%	S_R_<2·S_r_
	LR EH	1.84[1.65, 2.029]	0.42	0.42	22.83%	S_R_<2·S_r_
	LR CA	1.80[1.564, 2.035]	0.53	0.36	28.89%	S_R_<2·S_r_
	*R*I-TSA	3.35[3.275, 3.418]	0.23	0.20	5.69%	S_R_<2·S_r_
	*R*I-MEA	3.20[3.078, 3.313]	0.39	0.29	11.88%	S_R_<2·S_r_
	*W*W-TSA	4.58[4.378, 4.775]	0.61	0.26	12.88%	4·S_r_>S_R_>2·S_r_
	*W*W-MEA	4.40[4.189, 4.615]	0.63	0.56	14.32%	S_R_<2·S_r_
TEST B	LR PA	3.13[2.518, 3.746]	1.40	0.68	43.13%	4·S_r_>S_R_>2·S_r_
	LR EC	3.61[3.037, 4.187]	1.32	0.49	34.90%	4·S_r_>S_R_>2·S_r_
	LR SA	2.27[1.772, 2.758]	1.04	0.69	44.93%	S_R_<2·S_r_
	LR EH	2.61[2.135, 3.083]	1.07	0.70	39.85%	S_R_<2·S_r_
	LR CA	2.13[1.839, 2.427]	0.67	0.36	30.05%	S_R_<2·S_r_
	*R*I-TSA	2.95[2.751, 3.139]	0.65	0.32	22.45%	4·S_r_>S_R_>2·S_r_
	*R*I-MEA	2.71[2.542, 2.877]	0.56	0.29	19.93%	S_R_<2·S_r_
	*W*W-TSA	3.29[3.11, 3.469]	0.41	0.19	11.85%	4·S_r_>S_R_>2·S_r_
	*W*W-MEA	3.37[3.206, 3.531]	0.38	0.04	10.68%	S_R_>4·S_r_
TEST C	LR PA	5.51[5.238, 5.779]	0.61	0.64	10.71%	S_R_<2·S_r_
	LR EC	4.91[4.615, 5.204]	0.68	0.24	13.27%	4·S_r_>S_R_>2·S_r_
	LR SA	5.05[4.829, 5.273]	0.51	0.13	9.70%	4·S_r_>S_R_>2·S_r_
	LR EH	4.85[4.541, 5.162]	0.71	0.43	13.20%	S_R_<2·S_r_
	LR CA	2.97[2.633, 3.315]	0.76	0.65	24.92%	S_R_<2·S_r_
	*R*I-TSA	1.72[1.615, 1.828]	0.35	0.24	20.35%	S_R_<2·S_r_
	*R*I-MEA	1.76[1.637, 1.886]	0.41	0.31	22.73%	S_R_<2·S_r_
	*W*W-TSA	1.92[1.592, 2.245]	0.74	0.38	22.22%	S_R_<2·S_r_
	*W*W-MEA	2.31[2.013, 2.615]	0.68	0.40	28.57%	S_R_<2·S_r_

**Variable:** Each of the parameters evaluated in the method**, Mean:** Mean value of all runs taken into account**, CI 95%: 95%** confidence interval, **S**_**R**_: Reproducibility standard deviation, **S**_**r**_: Repeatability standard deviation, **RSD %**: Relative standard deviation, **S**_**R**_**/S**_**r**_: Ratio between repeatability and reproducibility standard deviation, **test A**: Water, **test B**: 0.66% IEC-A, **test C**: 0.50% IEC-A+0.135% perborate+0.02% TAED, **LR**: Logarithmic reduction, **PA**: *P*. *aeruginosa*, **EC**: *E*. *coli*, **SA**: *S*. *aureus*, **EH**: *E*. *hirae*, **CA**: *C*. *albicans*, ***R*I**: Cross contamination carrier, ***W*W**: Wash water, **TSA**: Trypticase soy agar, **MEA**: Malt extract agar.

**Table 2:** Precision statistics for testing per prEN17658 in the rinse cycle conditions.

	VARIABLE	Mean(CI95%)	S_R_		S_r_	RSD %	S_R_/S_r_
TEST D	LR PA	1.86[1.699, 2.029]		0.33	0.31	17.47%	S_R_<2·S_r_
	LR EC	1.501.267, 1.736]		0.48	0.39	31.33%	S_R_<2·S_r_
	LR SA	0.92[0.692, 1.147]		0.46	0.46	50.00%	S_R_<2·S_r_
	LR EH	1.38[1.182, 1.576]		0.40	0.38	28.99%	S_R_<2·S_r_
	LR CA	1.64[1.335, 1.953]		0.62	0.62	37.80%	S_R_<2·S_r_
	*R*I-TSA	3.60[3.466, 3.723]		0.40	0.21	9.58%	S_R_<2·S_r_
	*R*I-MEA	2.91[2.683, 3.141]		0.71	0.45	23.35%	S_R_<2·S_r_
	*W*W-TSA	4.84[4.627, 5.045]		0.78	0.20	12.19%	4·S_r_>S_R_>2·S_r_
	*W*W-MEA	4.29[4.037, 4.539]		0.85	0.18	16.55%	4·S_r_>S_R_>2·S_r_
TEST E	LR PA	2.63[2.189, 3.07]		0.91	0.71	33.84%	S_R_<2·S_r_
	LR EC	3.13[2.629, 3.645]		1.07	0.55	32.48%	S_R_<2·S_r_
	LR SA	3.06[2.533, 3.589]		1.12	0.44	34.64%	4·S_r_>S_R_>2·S_r_
	LR EH	2.94[2.349, 3.523]		1.23	0.66	40.14%	S_R_<2·S_r_
	LR CA	3.14[2.661, 3.624]		1.00	0.73	30.89%	S_R_<2·S_r_
	*R*I-TSA	2.02[1.789, 2.246]		0.70	0.50	31.22%	S_R_<2·S_r_
	*R*I-MEA	1.88[1.68, 2.071]		0.60	0.41	30.85%	4·S_r_>S_R_>2·S_r_
	*W*W-TSA	1.45[1.154, 1.748]		0.60	0.57	41.35%	4·S_r_>S_R_>2·S_r_
	*W*W-MEA	1.38[1.086, 1.672]		0.60	0.54	42.75%	4·S_r_>S_R_>2·S_r_
TEST F	LR PA	5.16[4.729, 5.595]		0.78	0.78	15.12%	S_R_<2·S_r_
	LR EC	5.06[4.738, 5.376]		0.58	0.58	11.46%	S_R_<2·S_r_
	LR SA	5.11[4.605, 5.619]		0.91	0.91	17.81%	S_R_<2·S_r_
	LR EH	5.00[4.482, 5.514]		0.94	0.87	18.60%	S_R_<2·S_r_
	LR CA	4.27[4.001, 4.539]		0.49	0.49	11.48%	S_R_<2·S_r_
	*R*I-TSA	1.61[1.527, 1.695]		0.23	0.19	15.53%	S_R_<2·S_r_
	*R*I-MEA	1.55[1.533, 1.56]		0.04	0.04	2.59%	S_R_<2·S_r_
	*W*W-TSA	1.15[1.15, 1.15]		0.00	0.00	0.00%	S_R_<2·S_r_
	*W*W-MEA	1.15[1.15, 1.15]		0.00	0.00	0.00%	S_R_<2·S_r_

**Variable:** Each of the parameters evaluated in the method**, Mean:** Mean value of all runs taken into account**, CI 95%:** 95% confidence interval, **S**_**R**_: Reproducibility standard deviation, **S**_**r**_: Repeatability standard deviation, **RSD** %: Relative standard deviation, **S**_**R**_**/S**_**r**_: Ratio between repeatability and reproducibility standard deviation, **test A**: Water, **test B**:0.04% DDAC, **test C**: 0.4% DDAC, **LR**: Logarithmic reduction, **PA**: *P*. *aeruginosa*, **EC**: *E*. *coli*, **SA**: *S*. *aureus*, **EH**: *E*. *hirae*, **CA**: *C*. *albicans*, ***R*I**: Cross-contamination carrier, ***W*W**: Wash water, **TSA**: Trypticase soy agar, **MEA**: Malt extract agar.

The results indicate that the method is sensitive to disinfectant concentration. Therefore, the higher the dose of active ingredient (tests C, D), the greater the reduction in *N*_a_ and the lower the recovery in *R*I and *W*W. This behaviour was observed in both test conditions (main wash and rinse cycles). Regarding *R*I, whereas little cross-contamination was detectable when the disinfectant was used (tests C, F), a significant transfer of microbial load could be observed in the test with the detergent alone (test B) and with the 0.04% DDAC dose (test E). A preventive effect of disinfectant-containing detergents has been previously reported in other studies related to household laundering processes [2,4,28]. Cross-contamination is a highly relevant parameter when evaluating the risk of infection and the probability of generating bad odours in textiles or washing machines [3,29,30–33]. In general, high levels of cross-contamination in water controls at a low temperature (20°C) have been previously reported [2].

According to the results generated by the interlaboratory work, the mechanical effect at 30 min and 30° C (test A, only water) reduces the level of microorganisms attached to the carriers by an average of 2.37 log_10_ (LR-PA, Table 1). Considering that *N*_0_ must have an initial microbial load of at least 6.15 log_10_, when a disinfectant is evaluated with this methodology, there is room for more than 4 log_10_ to demonstrate its inactivating effect during the laundering process.

Regarding the number of microbial cells and the composition of microbiota on textiles in daily life scenarios, no specific investigations have been done previously, but some studies suggest that the microbial count after normal use might be in the range of 10^2^−10^4^ cfu/cm^2^ [32,34]. Therefore, the present method is adapted to the conditions that can be found in real life.

### General results for S_R_ and S_r_

In the present method, disinfectant efficacy is measured by the LR of viable cells on the carriers and microorganism recovery (*R*I and *W*W), and good reproducibility is demonstrated when these values observed in the interlaboratory study are judged to be sufficiently similar. To help with that judgment, the variability among LR, *R*I and *W*W values was quantified by calculating the reproducibility standard deviation (S_R_) of these parameters. Decisions about the acceptability of an S_R_ often rely on historical precedent, i.e., the S_R_ values for disinfectant test methods already accepted by the community of experts. In their review of standard quantitative antimicrobial methods, Tilt and Hamilton [35] describe that S_R_ values range from 0.3 to 1.5, with a median of 0.9. The Montana State Center for Biofilm Engineering reports that an S_R_ ≤1.3or ≤1.5 is acceptable for most practical purposes. In the present study, reproducibility for all test products and conditions was high and acceptable according to the previous argumentation (all S_R_ values were between 0.00 and 1.40).

These results reflect that reproducibility is highest when the tests are carried out with water (tests A and D), or when the concentration of the disinfectant product is effective (tests C and F). All S_r_ values were below 1 (highest value = 0.91 corresponding to LR-SA test F), which expert judgement considers acceptable, given that there is no previous data available for this parameter (personal communication from experts Gebel J, Gemein S).

When the product is not effective, such as the detergent (test B), or is borderline effective (test E), the deviations are usually greater than for water or a disinfectant with a high efficacy. Even in those cases, the S_R_ exceeded a value of 1 only in LR-PA test B (S_R_ = 1.40) and LR-EH test E (S_R_ = 1.23) parameters (see Tables 1 and 2). Accordingly, it can be stated that the method is robust.

### Relative standard deviation (%RSD)

63% of the determinations were below 30%RSD, and no values were higher than 50%RSD (see Tables 1 and 2). The %RSD is also in line with other ring trial studies in which values below 30–50% indicate a satisfactory reproducibility.

### Outliers

In addition, an analysis was carried out using the Cochran test to detect if there is greater than normal variability [25] and the Grubbs test to detect outliers in the observations for each variable [26]. Of the 1017 values obtained during the intercalibration exercise, only 18 individual values were outliers (see annex, S1 and S2 Tables). When the statistical analyses were carried out again, no significant differences were detected (S3 and S4 Tables).

### Ratio between repeatability and reproducibility standard deviation (S_R_/S_r_)

In 74.1% of the comparisons, only minor systematic differences between laboratories were found (SR<2·Sr). In 24.1%, clear systematic differences between laboratories were observed (4·S_r_>S_R_>2·S_r_), but they were acceptable according to ISO 5725–2 recommendations, and considerable systematic differences (S_R_>4·S_r_) were found only in one comparison (test B, *W*W-MEA). Nevertheless, as it refers to a parameter in a low-medium efficacy concentration, it can be considered irrelevant.

### Sensitivity analysis: Effect of laboratory-scale device

**Fig 2:**
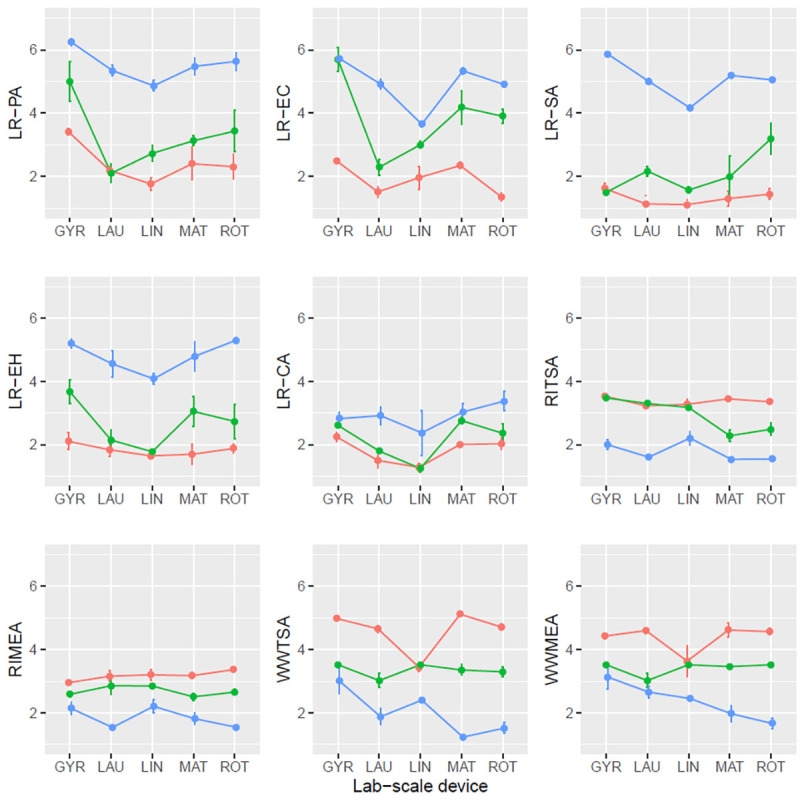
Mean plot with standard error bars of the different variables for main wash. Lines in blue: Concentration A; lines in green: Concentration B; lines in red: Concentration C. **LR**: Logarithmic reduction, **PA**: *P*. *aeruginosa*, **EC**: *E*. *coli*, **SA**: *S*. *aureus*, **EH**: *E*. *hirae*, **CA**: *C*. *albicans*, ***R*I**: Cross-contamination carrier, ***W*W**: Wash water, **TSA**: Trypticase soy agar, **MEA**: Malt extract agar, **GYR**: Gyrowash (James heal), **LAU**: Launderometer (SDL Atlas), **LIN**: Linitest (SDL Atlas), **MAT**: Labomat BFA-24 (Mathis AG).

**Fig 3:**
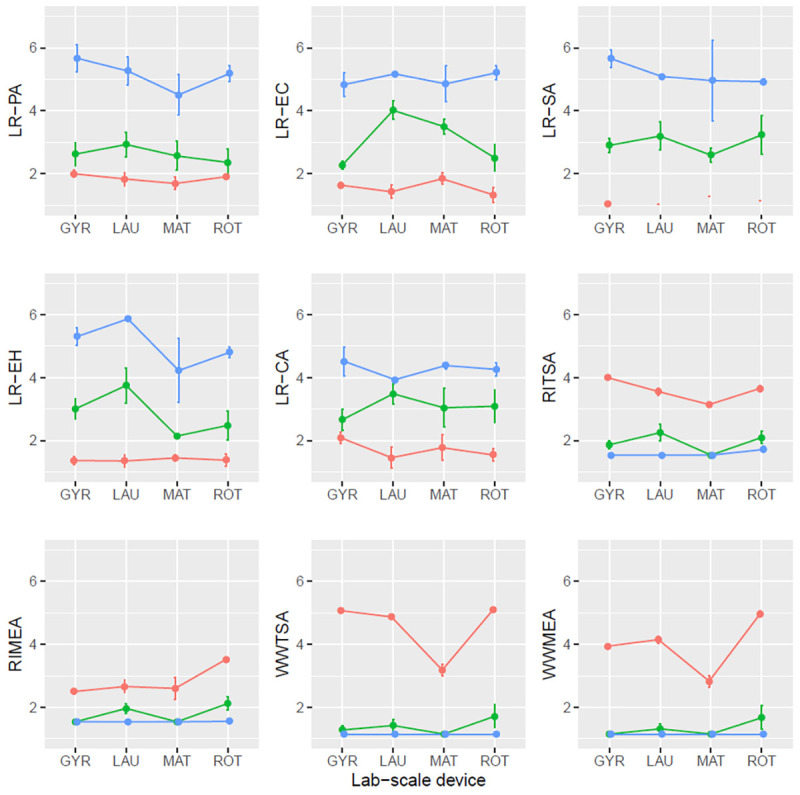
Mean plot with standard error bars of the different variables for the rinse cycle. **LR**: Logarithmic reduction, **PA**: *P*. *aeruginosa*, **EC**: *E*. *coli*, **SA**: *S*. *aureus*, **EH**: *E*. *hirae*, **CA**: *C*. *albicans*, ***R*I**: Cross- contamination carrier, ***W*W**: Wash water, **TSA**: Trypticase soy agar, **MEA**: Malt extract agar, **GYR**: Gyrowash (James heal), **LAU**: Launderometer (SDL Atlas), **LIN**: Linitest (SDL Atlas), **MAT**: Labomat BFA-24 (Mathis AG). Lines in blue: Concentration D; lines in green: Concentration E; lines in red: Concentration F.

An effect of the laboratory-scale device was detected only for LR-EC and LR-CA, although the magnitude was not significant (see Figs 2 and 3). This effect is not possible to separate from the effect of the laboratories [confusion of factors: two laboratory-scale devices (Rotawash and Launderometer) were used in more than one laboratory and the rest only in one laboratory].

## Conclusion

The new method (prEN 17658), carried out in a laboratory-scale washing machine, not only simulates domestic laundry processes but is sufficiently robust to be applied as a standard. Testing the efficacy of laundry disinfection is a complex process involving a range of parameters. Thus, a method that allows all the parameters (temperature, contact time, mechanical effect, and chemistry) to be controlled separately provides more consistent reproducibility, as demonstrated here. The method also reproduces domestic laundering conditions in a more realistic way compared to existing protocols in which antimicrobial products are evaluated in industrial washing machines with markedly different characteristics. Moreover, previous studies indicate that a laboratory-scale device is not significantly different from a domestic washing machine, while having an important advantage in terms of reproducibility and repeatability [2,9].

This new methodology has improved capacity and costs compared to the current protocol (EN16616), in which each microorganism is tested separately and only one test can be performed per washing machine. In contrast, the new protocol allows for up to 20 tests to be carried out simultaneously (depending on the laboratory-scale device) and all microorganisms are tested in the same run.

## Supporting information

S1 Fig Main wash and rinsing cycle canister distribution. I: Main wash canister, II: Rinsing cycle canister, A: inoculated carriers, B: sterile carriers, C: steel beads; D: ballast load, E: interference substance (SBL2004).
(TIF)

S1 Table Precision statistics for testing per prEN 17658 in the main wash conditions. Results of all the estimates of the variance components, the Cochran test for the detection of abnormal variance and the Grubbs test for the detection of outliers are shown.
(DOCX)

S2 Table Precision statistics for testing per pr EN17658 in the rinse cycle conditions. Results of all the estimates of the variance components, the Cochran test for the detection of abnormal variance and the Grubbs test for the detection of outliers are shown.
(DOCX)

S3 Table Precision statistics for testing per prEN 17658 in the main wash conditions. Removing the outliers.
(DOCX)

S4 Table Precision statistics for testing pr EN17658 in the rinse cycle conditions. Removing the outliers.
(DOCX)

S5 Table Experimental design. Test products and corresponding neutralizer used. **Neutralizer 1**: 20 g/l sodium thiosulfate; 30 g/l polysorbate 80; 3 g/l lecithin; 1 g/l L-histidine; 30 g/l saponin. **Neutralizer 2**: 5 g/l casein enzymatic hydrolysate; 2.50 g/l yeast extract; 10 g/l dextrose; 6 g/l sodium thiosulfate; 1 g/l sodium thioglycolate; 2.5 g/l sodium bisulfite; 7 g/l lecithin; 5 g/l polysorbate 80; 0,02 g/l bromocresol purple. **Neutralizer 3**: 17 g/l casein peptone; 3 g/l soy peptone; 5 g/l sodium chloride; 2.5 g/l phosphate buffer; 2,5 g/l glucose monohydrate; 3 g/l lecithin; 30 ml/ polysorbate; 1 g/l histidine; 5 g/l sodium thiosulfate. **Neutralizer 4**: 10 g/l sodium thiosulfate; 60 g/l polysorbate 80; 9 g/l lecithin; 4 g/l L-histidine; 60 g/l saponin; 4 g/l sodium dodecyl sulphate. **Neutralizer 5**: 3 g/l lecithin; 5 g/l sodium thiosulfate; 1 g/l L-histidine; 1 g/l casein peptone; 8,5g sodium chloride; 1 g/l dipotassium phosphate. **Neutralizer 6**: 3 g/l lecithin; 30 ml/l polysorbate 80; 5 g/l sodium thiosulfate; 1 g/l L-histidine; 30 g/l saponin, **test A**: water, **test B**: 0.66% IEC-A, **test C**: 0.50% IEC-A+0.135% perborate+0.02% TAED, **test D**: water, **test E**:0,04% DDAC, **test F**: 0,4% DDAC.
(DOCX)

S6 Table *N*_0_ compilation values. Compilation of all *N*_0_ values of the study. **Mean value**: mean value of all *N*_0_ for each strain. **sd**: standard deviation of all *N*_0_ for each strain, **PA**: *Pseudomona aeruginosa*, **EC**: *Escherichia coli*, **SA**: *Staphylococcus aureus*, **EH**: *Enterococcus hirae*, **CA**: *Candida albicans*.
(DOCX)
