# Supplementary figures and images for: International ring trial to validate a new method for testing the antimicrobial efficacy of domestic laundry products
Source: PLoS One. 2022 Jun 3;17(6):e0269556. doi: 10.1371/journal.pone.0269556 (PMC9165900; doi:10.1371/journal.pone.0269556)

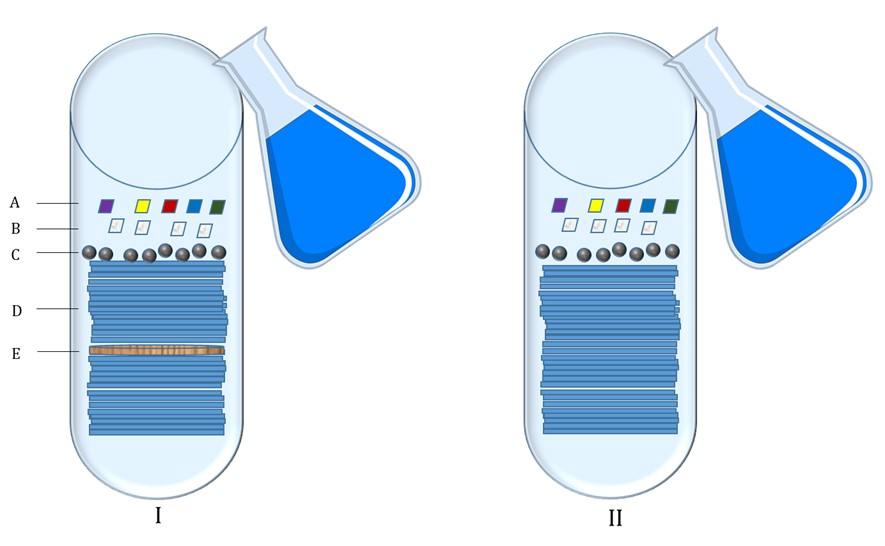

Supplement: S1 Fig — I: Main wash canister, II: Rinsing cycle canister, A: inoculated carriers, B: sterile carriers, C: steel beads; D: ballast load, E: interference substance (SBL2004). (TIF) [file pone.0269556.s001.tif]
