# Supplementary material for: International ring trial to validate a new method for testing the antimicrobial efficacy of domestic laundry products
Source: PLoS One. 2022 Jun 3;17(6):e0269556. doi: 10.1371/journal.pone.0269556 (PMC9165900; doi:10.1371/journal.pone.0269556)
Supplement: S1 Table — Results of all the estimates of the variance components, the Cochran test for the detection of abnormal variance and the Grubbs test for the detection of outliers are shown. (DOCX) [file pone.0269556.s002.docx]

**Table S1. Precision statistics for testing per prEN 17658 in the main wash conditions. Results of all the estimates of the variance components, the Cochran test for the detection of abnormal variance and the Grubbs test for the detection of outliers are shown.**

|  | **VARIABLE** | **S_R_** | **S_r_** | **S_B_**  **(p-value)** | **Cochran test outlier detection** | **Grubbs test individual outliers** |
| --- | --- | --- | --- | --- | --- | --- |
| TEST A | LR PA | 0.77 | 0.58 | 0.52* |  |  |
|  | LR EC | 0.59 | 0.31 | 0.49*** |  |  |
|  | LR SA | 0.48 | 0.36 | 0.33* |  |  |
|  | LR EH | 0.42 | 0.42 | 0 |  |  |
|  | LR CA | 0.53 | 0.36 | 0.39** |  |  |
|  | *R*I-TSA | 0.23 | 0.20 | 0.13** |  | * |
|  | *R*I-MEA | 0.39 | 0.29 | 0.26*** |  | * |
|  | *W*W-TSA | 0.61 | 0.26 | 0.55*** |  |  |
|  | *W*W-MEA | 0.63 | 0.56 | 0.31* |  | * |
| TEST B | LR PA | 1.40 | 0.68 | 1.23*** | *** |  |
|  | LR EC | 1.32 | 0.49 | 1.23*** | *** |  |
|  | LR SA | 1.04 | 0.69 | 0.77* |  |  |
|  | LR EH | 1.07 | 0.70 | 0.81** |  |  |
|  | LR CA | 0.67 | 0.36 | 0.57** |  |  |
|  | *R*I-TSA | 0.65 | 0.32 | 0.56*** |  |  |
|  | *R*I-MEA | 0.56 | 0.29 | 0.48*** | *** | * |
|  | *W*W-TSA | 0.41 | 0.19 | 0.36*** |  |  |
|  | *W*W-MEA | 0.38 | 0.04 | 0.37*** |  |  |
| TEST C | LR PA | 0.61 | 0.64 | 0.45** |  |  |
|  | LR EC | 0.68 | 0.24 | 0.63*** |  |  |
|  | LR SA | 0.51 | 0.13 | 0.50*** |  |  |
|  | LR EH | 0.71 | 0.43 | 0.57** |  |  |
|  | LR CA | 0.76 | 0.65 | 0.40 |  |  |
|  | *R*I-TSA | 0.35 | 0.24 | 0.25*** |  |  |
|  | *R*I-MEA | 0.41 | 0.31 | 0.27*** |  |  |
|  | *W*W-TSA | 0.74 | 0.38 | 0.64*** |  |  |
|  | *W*W-MEA | 0.68 | 0.40 | 0.56*** |  |  |

**LR**: Logarithmic reduction, **PA**: *P. aeruginosa*, **EC**: *E. coli*, **SA**: *S. aureus*, **EH**: *E. hirae*, **CA**: *C. albicans,* ***R*I**: cross-contamination carrier, ***W*W**: wash water, **TSA**: trypticase soy agar, **MEA**: malt extract agar, **test A**: water, **test B**: 0.66% IEC-A, **test C**: 0.50% IEC-A+0.135% perborate+0.02% TAED.

Column S_B_ presents estimation of $\boldsymbol{\sigma}_{\boldsymbol{B}}$ and p-value corresponding to ANOVA test $\boldsymbol{\sigma}_{\boldsymbol{B}}^{\boldsymbol{2}}$ > 0.

Statistical test p-values: * p<0.05, ** p<0.01, *** p<0.001.
