# Supplementary material for: International ring trial to validate a new method for testing the antimicrobial efficacy of domestic laundry products
Source: PLoS One. 2022 Jun 3;17(6):e0269556. doi: 10.1371/journal.pone.0269556 (PMC9165900; doi:10.1371/journal.pone.0269556)
Supplement: S2 Table — Results of all the estimates of the variance components, the Cochran test for the detection of abnormal variance and the Grubbs test for the detection of outliers are shown. (DOCX) [file pone.0269556.s003.docx]

**Table S2. Precision statistics for testing per pr EN17658 in the rinse cycle conditions. Results of all the estimates of the variance components, the Cochran test for the detection of abnormal variance and the Grubbs test for the detection of outliers are shown.**

|  | **VARIABLE** | **S_R_** | **S_r_** | **S_B_**  **(p-value)** | | **Cochran test variability higher than expected** | **Grubbs test individual outliers** |
| --- | --- | --- | --- | --- | --- | --- | --- |
| TEST D | LR PA | 0.33 | 0.31 | 0.13 |  | |  |
|  | LR EC | 0.48 | 0.39 | 0.29 |  | |  |
|  | LR SA | 0.46 | 0.46 | 0 |  | |  |
|  | LR EH | 0.40 | 0.38 | 0.11 |  | |  |
|  | LR CA | 0.62 | 0.62 | 0 |  | |  |
|  | RI-TSA | 0.40 | 0.21 | 0.34* |  | |  |
|  | RI-MEA | 0.71 | 0.45 | 0.54*** | *** | |  |
|  | *W*W-TSA | 0.78 | 0.20 | 0.76*** |  | |  |
|  | *W*W-MEA | 0.85 | 0.18 | 0.83*** |  | |  |
| TEST E | LR PA | 0.91 | 0.71 | 0.56 |  | |  |
|  | LR EC | 1.07 | 0.55 | 0.92 * | ** | |  |
|  | LR SA | 1.12 | 0.44 | 1.03* |  | |  |
|  | LR EH | 1.23 | 0.66 | 1.04* | * | |  |
|  | LR CA | 1.00 | 0.73 | 0.68* | * | |  |
|  | *R*I-TSA | 0.70 | 0.50 | 0.49* | *** | |  |
|  | *R*I-MEA | 0.60 | 0.41 | 0.45*** |  | |  |
|  | *W*W-TSA | 0.60 | 0.57 | 0.19 |  | | * |
|  | *W*W-MEA | 0.60 | 0.54 | 0.26 |  | | *** |
| TEST F | LR PA | 0.78 | 0.78 | 0 |  | | * |
|  | LR EC | 0.58 | 0.58 | 0 |  | |  |
|  | LR SA | 0.91 | 0.91 | 0 |  | | *** |
|  | LR EH | 0.94 | 0.87 | 0.36 |  | | *** |
|  | LR CA | 0.49 | 0.49 | 0 |  | |  |
|  | *R*I-TSA | 0.23 | 0.19 | 0.14*** |  | | *** |
|  | *R*I-MEA | 0.04 | 0.04 | 0 |  | | *** |
|  | *W*W-TSA | 0.00 | 0.00 | 0 |  | | *** |
|  | *W*W-MEA | 0.00 | 0.00 | 0 |  | |  |

**LR**: Logarithmic reduction, **PA**: *P. aeruginosa*, **EC**: *E. coli*, **SA**: *S. aureus*, **EH**: *E. hirae*, **CA**: *C. albicans,* ***R*I**: cross-contamination carrier, ***W*W**: wash water, **TSA**: trypticase soy agar, **MEA**: malt extract agar, **test D**: water, **test E**:0,04% DDAC, **test F**: 0,4% DDAC.

Column S_B_ presents estimation of $\boldsymbol{\sigma}_{\boldsymbol{B}}$ and p-value corresponding to ANOVA test $\boldsymbol{\sigma}_{\boldsymbol{B}}^{\boldsymbol{2}}$ > 0: * p<0.05, ** p<0.01, *** p<0.001.

Cochran test: * p<0.05, ** p<0.01, *** p<0.001.

Tables S1and S2 provide additional information about the decomposition of variability (SB) and Cochran test analyses to detect outliers and higher variability than expected. Regarding the significant variability “between laboratory”$(\boldsymbol{\sigma}_{\boldsymbol{B}}^{\boldsymbol{2}}$ > 0) it should be noted that the hypothesis test on $\boldsymbol{\sigma}_{\boldsymbol{B}}^{\boldsymbol{2}}$ refers to whether or not this parameter is $\boldsymbol{\sigma}_{\boldsymbol{B}}^{\boldsymbol{2}}$ > 0, not the magnitude of $\boldsymbol{\sigma}_{\boldsymbol{B}}^{\boldsymbol{2}}$ . Given that none of the comparisons exceed the established limits, the method robustness is proved.
