# Supplementary material for: International ring trial to validate a new method for testing the antimicrobial efficacy of domestic laundry products
Source: PLoS One. 2022 Jun 3;17(6):e0269556. doi: 10.1371/journal.pone.0269556 (PMC9165900; doi:10.1371/journal.pone.0269556)
Supplement: S3 Table — Removing the outliers. (DOCX) [file pone.0269556.s004.docx]

**Table S3. Precision statistics for testing per prEN 17658 in the main wash conditions. Removing the outliers.**

|  | **VARIABLE** | **Mean(CI95%)** | **S_R_** | **S_r_** | **SB** | **p-value ANOVA** |
| --- | --- | --- | --- | --- | --- | --- |
| TEST A | LR PA | 2.37[2.026, 2.714] | 0.60 | 0.33 | 0.27 | * |
|  | LR EC | 1.79[1.534, 2.048] | 0.34 | 0.10 | 0.24 | *** |
|  | LR SA | 1.31[1.097, 1.527] | 0.23 | 0.13 | 0.11 | * |
|  | LR EH | 1.84[1.65, 2.029] | 0.17 | 0.17 | 0.00 |  |
|  | LR CA | 1.8[1.564, 2.035] | 0.28 | 0.13 | 0.15 | ** |
|  | *R*I-TSA | 3.33[3.265, 3.395] | 0.05 | 0.02 | 0.03 | *** |
|  | *R*I-MEA | 3.22[3.117, 3.329] | 0.12 | 0.07 | 0.05 | *** |
|  | *W*W-TSA | 4.58[4.378, 4.775] | 0.37 | 0.07 | 0.30 | *** |
|  | *W*W-MEA | 4.49[4.323, 4.65] | 0.22 | 0.19 | 0.04 |  |
| TEST B | LR PA | 3.13[2.518, 3.746] | 1.40 | 0.68 | 1.23 | *** |
|  | LR EC | 3.61[3.037, 4.187] | 1.32 | 0.49 | 1.23 | *** |
|  | LR SA | 2.27[1.772, 2.758] | 1.04 | 0.69 | 0.77 | * |
|  | LR EH | 2.61[2.135, 3.083] | 1.07 | 0.70 | 0.81 | ** |
|  | LR CA | 2.17[1.859, 2.486] | 0.67 | 0.19 | 0.64 | *** |
|  | *R*I-TSA | 2.95[2.751, 3.139] | 0.65 | 0.32 | 0.56 | *** |
|  | *R*I-MEA | 2.65[2.502, 2.795] | 0.51 | 0.26 | 0.44 | *** |
|  | *W*W-TSA | 3.33[3.132, 3.519] | 0.41 | 0.12 | 0.39 | *** |
|  | *W*W-MEA | 3.35[3.163, 3.544] | 0.23 | 0.00 | 0.23 | *** |
| TEST C | LR PA | 5.51[5.238, 5.779] | 0.61 | 0.41 | 0.45 | ** |
|  | LR EC | 4.94[4.602, 5.273] | 0.72 | 0.16 | 0.70 | *** |
|  | LR SA | 5.05[4.829, 5.273] | 0.51 | 0.13 | 0.50 | *** |
|  | LR EH | 4.85[4.541, 5.162] | 0.71 | 0.43 | 0.57 | ** |
|  | LR CA | 2.97[2.633, 3.315] | 0.76 | 0.65 | 0.40 |  |
|  | *R*I-TSA | 1.64[1.564, 1.717] | 0.23 | 0.16 | 0.17 | *** |
|  | *R*I-MEA | 1.69[1.576, 1.799] | 0.34 | 0.26 | 0.22 | ** |
|  | *W*W-TSA | 1.92[1.592, 2.245] | 0.74 | 0.38 | 0.64 | ** |
|  | *W*W-MEA | 2.31[2.013, 2.615] | 0.68 | 0.40 | 0.56 | ** |

**LR**: Logarithmic reduction, **PA**: *P. aeruginosa*, **EC**: *E. coli*, **SA**: *S. aureus*, **EH**: *E. hirae*, **CA**: *C. albicans,* ***R*I**: cross-contamination carrier, ***W*W**: wash water, **TSA**: trypticase soy agar, **MEA**: malt extract agar, **test A**: water, **test B**: 0.66% IEC-A, **test C**: 0.50% IEC-A+0.135% perborate+0.02% TAED.

Column p-value ANOVA presents p-value corresponding to ANOVA test $\boldsymbol{\sigma}_{\boldsymbol{B}}^{\boldsymbol{2}}$ > 0: * p<0.05, ** p<0.01, *** p<0.001.
