# Supplementary material for: International ring trial to validate a new method for testing the antimicrobial efficacy of domestic laundry products
Source: PLoS One. 2022 Jun 3;17(6):e0269556. doi: 10.1371/journal.pone.0269556 (PMC9165900; doi:10.1371/journal.pone.0269556)
Supplement: S4 Table — Removing the outliers. (DOCX) [file pone.0269556.s005.docx]

**Table S4. Precision statistics for testing pr EN17658 in the rinse cycle conditions. Removing the outliers.**

|  | **VARIABLE** | **Mean(CI95%)** | **S_R_** | | **S_r_** | **RSD %** | **S_R_/S_r_** |
| --- | --- | --- | --- | --- | --- | --- | --- |
| TEST D | LR PA | 1.86[1.699, 2.029] | | 0.11 | 0.10 | 0.02 |  |
|  | LR EC | 1.50[1.267, 1.736] | | 0.23 | 0.15 | 0.08 |  |
|  | LR SA | 0.92[0.692, 1.147] | | 0.21 | 0.21 | 0.00 |  |
|  | LR EH | 1.38[1.182, 1.576] | | 0.16 | 0.15 | 0.01 |  |
|  | LR CA | 1.69[1.416, 1.963] | | 0.26 | 0.17 | 0.08 |  |
|  | *R*I-TSA | 3.60[3.466, 3.723] | | 0.16 | 0.05 | 0.12 | *** |
|  | *R*I-MEA | 2.97[2.734, 3.213] | | 0.47 | 0.11 | 0.36 | *** |
|  | *W*W-TSA | 5.00[4.906, 5.097] | | 0.07 | 0.03 | 0.04 | *** |
|  | *W*W-MEA | 4.29[4.037, 4.539] | | 0.72 | 0.03 | 0.69 | *** |
| TEST E | LR PA | 2.63[2.189, 3.07] | | 0.91 | 0.71 | 0.56 |  |
|  | LR EC | 3.14[2.629, 3.645] | | 1.07 | 0.55 | 0.92 | *** |
|  | LR SA | 3.06[2.533, 3.589] | | 1.12 | 0.44 | 1.03 | *** |
|  | LR EH | 2.94[2.349. 3.523] | | 1.23 | 0.66 | 1.04 | ** |
|  | LR CA | 3.14[2.661, 3.624] | | 1.00 | 0.73 | 0.68 | * |
|  | *R*I-TSA | 1.87[1.678, 2.071] | | 0.55 | 0.37 | 0.42 | *** |
|  | *R*I-MEA | 1.71[1.562, 1.858] | | 0.41 | 0.33 | 0.24 | ** |
|  | *W*W-TSA | 1.27[1.168, 1.368] | | 0.19 | 0.19 | 0.00 |  |
|  | *W*W-MEA | 1.18[1.118, 1.238] | | 0.10 | 0.00 | 0.10 | *** |
| TEST F | LR PA | 5.30[4.956, 5.643] | | 0.59 | 0.59 | 0.00 |  |
|  | LR EC | 5.06[4.738, 5.376] | | 0.58 | 0.58 | 0.00 |  |
|  | LR SA | 5.31[4.992, 5.619] | | 0.61 | 0.27 | 0.54 | ** |
|  | LR EH | 5.20[4.893, 5.506] | | 0.54 | 0.38 | 0.39 | * |
|  | LR CA | 4.27[4.001, 4.539] | | 0.49 | 0.49 | 0.00 |  |
|  | *R*I-TSA | 1.57[1.514, 1.632] | | 0.20 | 0.10 | 0.17 | *** |
|  | *R*I-MEA | 1.54[1.54, 1.54] | | 0.00 | 0.00 | 0.00 |  |
|  | *W*W-TSA | 1.15[1.15, 1.15] | | 0.00 | 0.00 | 0.00 |  |
|  | *W*W-MEA | 1.15[1.15, 1.15] | | 0.00 | 0.00 | 0.00 |  |

**LR**: Logarithmic reduction, **PA**: *P. aeruginosa*, **EC**: *E. coli*, **SA**: *S. aureus*, **EH**: *E. hirae*, **CA**: *C. albicans,* ***R*I**: cross-contamination carrier, ***W*W**: wash water, **TSA**: trypticase soy agar, **MEA**: malt extract agar, **test D**: water, **test E**:0,04% DDAC, **test F**: 0,4% DDAC.

Column p-value ANOVA presents p-value corresponding to ANOVA test $\boldsymbol{\sigma}_{\boldsymbol{B}}^{\boldsymbol{2}}$ > 0: * p<0.05, ** p<0.01, *** p<0.001

Tables S3and S4 show the results for precision statistics when the outliers detected with the Grubb test were removed. As observed in the comparison table, the values of the estimated variance components, as well as the mean estimate and the CI95% were practically the same.
