# Supplementary material for: International ring trial to validate a new method for testing the antimicrobial efficacy of domestic laundry products
Source: PLoS One. 2022 Jun 3;17(6):e0269556. doi: 10.1371/journal.pone.0269556 (PMC9165900; doi:10.1371/journal.pone.0269556)
Supplement: S5 Table — Test products and corresponding neutralizer used. Neutralizer 1: 20 g/l sodium thiosulfate; 30 g/l polysorbate 80; 3 g/l lecithin; 1 g/l L-histidine; 30 g/l saponin. Neutralizer 2: 5 g/l casein enzymatic hydrolysate; 2.50 g/l yeast extract; 10 g/l dextrose; 6 g/l sodium thiosulfate; 1 g/l sodium thioglycolate; 2.5 g/l sodium bisulfite; 7 g/l lecithin; 5 g/l polysorbate 80; 0,02 g/l bromocresol purple. Neutralizer 3: 17 g/l casein peptone; 3 g/l soy peptone; 5 g/l sodium chloride; 2.5 g/l phosphate buffer; 2,5 g/l glucose monohydrate; 3 g/l lecithin; 30 ml/ polysorbate; 1 g/l histidine; 5 g/l sodium thiosulfate. Neutralizer 4: 10 g/l sodium thiosulfate; 60 g/l polysorbate 80; 9 g/l lecithin; 4 g/l L-histidine; 60 g/l saponin; 4 g/l sodium dodecyl sulphate. Neutralizer 5: 3 g/l lecithin; 5 g/l sodium thiosulfate; 1 g/l L-histidine; 1 g/l casein peptone; 8,5g sodium chloride; 1 g/l dipotassium phosphate. Neutralizer 6: 3 g/l lecithin; 30 ml/l polysorbate 80; 5 g/l sodium thiosulfate; 1 g/l L-histidine; 30 g/l saponin, test A: water, test B: 0.66% IEC-A, test C: 0.50% IEC-A+0.135% perborate+0.02% TAED, test D: water, test E:0,04% DDAC, test F: 0,4% DDAC. (DOCX) [file pone.0269556.s006.docx]

**Table S5. Experimental design.** Test products and corresponding neutralizer used. **Neutralizer 1**: 20 g/l sodium thiosulfate; 30 g/l polysorbate 80; 3 g/l lecithin; 1 g/l L-histidine; 30 g/l saponin. **Neutralizer 2**: 5 g/l casein enzymatic hydrolysate; 2.50 g/l yeast extract; 10 g/l dextrose; 6 g/l sodium thiosulfate; 1 g/l sodium thioglycolate; 2.5 g/l sodium bisulfite; 7 g/l lecithin; 5 g/l polysorbate 80; 0,02 g/l bromocresol purple. **Neutralizer 3**: 17 g/l casein peptone; 3 g/l soy peptone; 5 g/l sodium chloride; 2.5 g/l phosphate buffer; 2,5 g/l glucose monohydrate; 3 g/l lecithin; 30 ml/ polysorbate; 1 g/l histidine; 5 g/l sodium thiosulfate. **Neutralizer 4**: 10 g/l sodium thiosulfate; 60 g/l polysorbate 80; 9 g/l lecithin; 4 g/l L-histidine; 60 g/l saponin; 4 g/l sodium dodecyl sulphate. **Neutralizer 5**: 3 g/l lecithin; 5 g/l sodium thiosulfate; 1 g/l L-histidine; 1 g/l casein peptone; 8,5g sodium chloride; 1 g/l dipotassium phosphate. **Neutralizer 6**: 3 g/l lecithin; 30 ml/l polysorbate 80; 5 g/l sodium thiosulfate; 1 g/l L-histidine; 30 g/l saponin, **test A**: water, **test B**: 0.66% IEC-A, **test C**: 0.50% IEC-A+0.135% perborate+0.02% TAED, **test D**: water, **test E**:0,04% DDAC, **test F**: 0,4% DDAC.

| **Test code** | **A** | **B** | **C** | **D** | **E** | **F** |
| --- | --- | --- | --- | --- | --- | --- |
| **Active ingredient** | _ | 0.04% DDAC | 0.40% DDAC | _ | 0.66% IEC A | 0.66% IEC A 0.14% TAED 0.02% Perborate |
| **Neutralizer used** | | | | | | |
| **lab 1** | 5 | 5 | 4 | 4 | 4 | 4 |
| **lab 2** | 2 | 2 | 4 | 2 | 2 | 2 |
| **lab 3** | 3 | 3 | 4 | 4 | 4 | 4 |
| **lab 4** | 1 | 1 | 4 | 4 | 4 | 4 |
| **lab 5** | 6 | 6 | 4 | 6 | 6 | 6 |
| **lab 6** | nd | nd | nd | 3 | 3 | 3 |
| **lab 7** | 2 | 2 | 4 | 2 | 2 | 2 |
