# Supplementary material for: International ring trial to validate a new method for testing the antimicrobial efficacy of domestic laundry products
Source: PLoS One. 2022 Jun 3;17(6):e0269556. doi: 10.1371/journal.pone.0269556 (PMC9165900; doi:10.1371/journal.pone.0269556)
Supplement: S6 Table — Compilation of all N0 values of the study. Mean value: mean value of all N0 for each strain. sd: standard deviation of all N0 for each strain, PA: Pseudomona aeruginosa, EC: Escherichia coli, SA: Staphylococcus aureus, EH: Enterococcus hirae, CA: Candida albicans. (DOCX) [file pone.0269556.s007.docx]

**Table S6. *N*_0_ compilation values.** Compilation of all *N*_0_ values of the study. **Mean value**: mean value of all *N*_0_ for each strain. **sd**: standard deviation of all *N*_0_ for each strain, **PA***: Pseudomona aeruginosa*, **EC**: *Escherichia coli*, **SA**: *Staphylococcus aureus*, **EH**: *Enterococcus hirae*, **CA**: *Candida albicans*

| ***N*_0_-strain** | ***N*_0_-PA** | | | ***N*_0_-EC** | | | ***N*_0_-SA** | | | ***N*_0_-EH** | | | ***N*_0_-CA** | | |
| --- | --- | --- | --- | --- | --- | --- | --- | --- | --- | --- | --- | --- | --- | --- | --- |
| ***N*_0_ values** | 6.28 | 7.24 | 7.64 | 6.18 | 6.67 | 6.97 | 6.17 | 6.61 | 7.04 | 6.22 | 6.79 | 6.99 | 5.17 | 5.53 | 6.02 |
|  | 6.32 | 7.27 | 7.65 | 6.19 | 6.68 | 6.98 | 6.17 | 6.61 | 7.04 | 6.26 | 6.79 | 6.99 | 5.17 | 5.53 | 6.02 |
|  | 6.37 | 7.28 | 7.65 | 6.19 | 6.68 | 6.98 | 6.17 | 6.64 | 7.07 | 6.30 | 6.79 | 6.99 | 5.17 | 5.57 | 6.02 |
|  | 6.37 | 7.28 | 7.65 | 6.20 | 6.68 | 6.98 | 6.17 | 6.64 | 7.08 | 6.35 | 6.80 | 7.00 | 5.18 | 5.59 | 6.02 |
|  | 6.38 | 7.29 | 7.65 | 6.21 | 6.68 | 6.99 | 6.17 | 6.66 | 7.15 | 6.43 | 6.80 | 7.02 | 5.18 | 5.59 | 6.02 |
|  | 6.50 | 7.29 | 7.67 | 6.22 | 6.68 | 6.99 | 6.18 | 6.66 | 7.29 | 6.49 | 6.81 | 7.02 | 5.18 | 5.59 | 6.02 |
|  | 6.54 | 7.29 | 7.68 | 6.22 | 6.69 | 7.00 | 6.18 | 6.66 | 7.29 | 6.52 | 6.81 | 7.02 | 5.18 | 5.61 | 6.03 |
|  | 6.63 | 7.32 | 7.69 | 6.22 | 6.69 | 7.00 | 6.20 | 6.66 | 7.29 | 6.54 | 6.81 | 7.02 | 5.19 | 5.62 | 6.04 |
|  | 6.63 | 7.32 | 7.70 | 6.24 | 6.69 | 7.00 | 6.21 | 6.66 | 7.30 | 6.55 | 6.82 | 7.06 | 5.19 | 5.64 | 6.04 |
|  | 6.68 | 7.32 | 7.71 | 6.25 | 6.71 | 7.01 | 6.22 | 6.66 | 7.30 | 6.55 | 6.83 | 7.06 | 5.19 | 5.66 | 6.07 |
|  | 6.68 | 7.32 | 7.71 | 6.29 | 6.74 | 7.03 | 6.22 | 6.67 | 7.30 | 6.55 | 6.83 | 7.06 | 5.22 | 5.68 | 6.09 |
|  | 6.71 | 7.34 | 7.72 | 6.29 | 6.74 | 7.05 | 6.22 | 6.67 | 7.31 | 6.55 | 6.84 | 7.09 | 5.24 | 5.68 | 6.09 |
|  | 6.73 | 7.34 | 7.73 | 6.31 | 6.75 | 7.09 | 6.33 | 6.67 | 7.31 | 6.57 | 6.85 | 7.14 | 5.25 | 5.69 | 6.10 |
|  | 6.74 | 7.39 | 7.73 | 6.31 | 6.77 | 7.14 | 6.33 | 6.69 | 7.33 | 6.58 | 6.85 | 7.15 | 5.28 | 5.69 | 6.11 |
|  | 6.76 | 7.41 | 7.73 | 6.32 | 6.78 | 7.14 | 6.35 | 6.70 | 7.36 | 6.58 | 6.86 | 7.17 | 5.29 | 5.70 | 6.13 |
|  | 6.77 | 7.43 | 7.74 | 6.32 | 6.80 | 7.17 | 6.35 | 6.71 | 7.40 | 6.61 | 6.86 | 7.21 | 5.30 | 5.70 | 6.17 |
|  | 6.77 | 7.43 | 7.74 | 6.33 | 6.80 | 7.19 | 6.40 | 6.73 | 7.40 | 6.63 | 6.87 | 7.23 | 5.33 | 5.70 | 6.29 |
|  | 6.79 | 7.44 | 7.76 | 6.33 | 6.80 | 7.19 | 6.41 | 6.73 | 7.40 | 6.65 | 6.87 | 7.30 | 5.36 | 5.74 | 6.30 |
|  | 6.81 | 7.45 | 7.77 | 6.33 | 6.80 | 7.22 | 6.47 | 6.75 | 7.41 | 6.65 | 6.87 | 7.32 | 5.38 | 5.77 | 6.34 |
|  | 6.86 | 7.45 | 7.77 | 6.34 | 6.82 | 7.23 | 6.48 | 6.75 | 7.44 | 6.66 | 6.88 | 7.32 | 5.38 | 5.78 | 6.34 |
|  | 6.94 | 7.46 | 7.80 | 6.35 | 6.82 | 7.24 | 6.49 | 6.75 | 7.45 | 6.67 | 6.88 | 7.34 | 5.38 | 5.78 | 6.34 |
|  | 6.94 | 7.47 | 7.81 | 6.35 | 6.85 | 7.24 | 6.50 | 6.77 | 7.45 | 6.69 | 6.88 | 7.34 | 5.38 | 5.80 | 6.36 |
|  | 7.00 | 7.47 | 7.82 | 6.39 | 6.86 | 7.25 | 6.54 | 6.78 | 7.46 | 6.69 | 6.89 | 7.34 | 5.38 | 5.81 | 6.36 |
|  | 7.07 | 7.47 | 7.82 | 6.44 | 6.87 | 7.28 | 6.54 | 6.79 | 7.46 | 6.69 | 6.92 | 7.36 | 5.39 | 5.86 | 6.36 |
|  | 7.07 | 7.52 | 7.83 | 6.47 | 6.87 | 7.29 | 6.56 | 6.79 | 7.46 | 6.69 | 6.92 | 7.44 | 5.39 | 5.86 | 6.36 |
|  | 7.07 | 7.53 | 7.84 | 6.47 | 6.88 | 7.33 | 6.57 | 6.79 | 7.47 | 6.70 | 6.92 | 7.45 | 5.43 | 5.86 | 6.36 |
|  | 7.08 | 7.54 | 7.87 | 6.47 | 6.91 | 7.35 | 6.58 | 6.82 | 7.47 | 6.70 | 6.92 | 7.46 | 5.44 | 5.87 | 6.36 |
|  | 7.11 | 7.55 | 7.87 | 6.49 | 6.91 | 7.35 | 6.58 | 6.84 | 7.47 | 6.70 | 6.94 | 7.47 | 5.45 | 5.87 | 6.36 |
|  | 7.11 | 7.56 | 7.88 | 6.54 | 6.91 | 7.35 | 6.58 | 6.86 | 7.48 | 6.70 | 6.94 | 7.47 | 5.45 | 5.99 | 6.36 |
|  | 7.13 | 7.56 | 7.89 | 6.55 | 6.91 | 7.39 | 6.58 | 6.87 | 7.50 | 6.70 | 6.95 | 7.53 | 5.45 | 5.99 | 6.36 |
|  | 7.15 | 7.56 | 7.89 | 6.58 | 6.91 | 7.44 | 6.59 | 6.87 | 7.53 | 6.73 | 6.95 | 7.55 | 5.47 | 6.00 | 6.36 |
|  | 7.15 | 7.57 | 7.90 | 6.62 | 6.95 | 7.46 | 6.59 | 6.87 | 7.55 | 6.73 | 6.95 | 7.57 | 5.48 | 6.00 | 6.36 |
|  | 7.18 | 7.57 | 7.91 | 6.62 | 6.95 | 7.51 | 6.60 | 6.88 | 7.55 | 6.73 | 6.97 | 7.58 | 5.50 | 6.01 | 6.44 |
|  | 7.18 | 7.57 | 7.97 | 6.63 | 6.95 | 7.52 | 6.60 | 6.91 | 7.56 | 6.74 | 6.97 | 7.58 | 5.50 | 6.02 | 6.61 |
|  | 7.18 | 7.57 | 8.02 | 6.64 | 6.95 | 7.52 | 6.60 | 6.93 | 7.60 | 6.76 | 6.97 | 7.58 | 5.51 | 6.02 | 6.72 |
|  | 7.18 | 7.57 | 8.12 | 6.65 | 6.95 | 7.58 | 6.60 | 6.94 | 7.78 | 6.76 | 6.98 | 7.62 | 5.52 | 6.02 | 6.73 |
|  | 7.18 | 7.60 | 8.13 | 6.65 | 6.96 | 7.59 | 6.61 | 6.96 | 7.78 | 6.77 | 6.99 | 7.65 | 5.52 | 6.02 | 7.78 |
|  | 7.21 | 7.61 | 8.14 | 6.66 | 6.96 | 7.90 | 6.61 | 7.02 | 7.82 | 6.78 | 6.99 | 7.84 | 5.52 | 6.02 | 7.78 |
| **Mean value** | 7.36 | | | 6.81 | | | 6.86 | | | 6.93 | | | 5.82 | | |
| **sd** | 0.44 | | | 0.38 | | | 0.44 | | | 0.33 | | | 0.48 | | |

Table S5 shows the experimental design and Table S6 compiles all *N_0_* values of the study.
